# Switching Field Distribution in BN/FePtCAg/MgTiON and FePtCAg/MgTiOBN Films

**DOI:** 10.3390/nano12050874

**Published:** 2022-03-06

**Authors:** Jai-Lin Tsai, Chun-Yu Sun, Jun-Kai Lin, Gaun-Jhen Huang, Kuan-Cheng Liu, He-Ting Tsai

**Affiliations:** 1 Department of Materials Science and Engineering, National Chung Hsing University, Taichung 402, Taiwan; g109066021@mail.nchu.edu.tw (C.-Y.S.); g108066084@mail.nchu.edu.tw (J.-K.L.); g109066070@mail.nchu.edu.tw (G.-J.H.); g109066096@mail.nchu.edu.tw (K.-C.L.); 2 Instrument Center, The Office of Research & Development of Chung Hsing University, Taichung 402, Taiwan; httsai@nchu.edu.tw

**Keywords:** coercivity (normal to film surface), grain size, magnetocrystalline anisotropy, saturation magnetization, switch field distribution

## Abstract

BN is the currently required segregant for perpendicular FePt media. We found that BN can be diffused from the MgTiOBN intermediate layer during a high temperature process. The FePtCAg film sputtered on MgTiOBN layers illustrates higher perpendicular magnetocrystalline anisotropy (K_u_) (1.43 × 10^7^ erg/cm^3^) and coercivity (normal to film surface) (17 kOe) at 350 K compared to BN/FePtCAg/MgTiON film. From the microstructure, the FePtCAg film shows the granular structure on the MgTiOBN intermediate layer, but parts of the irregular FePt grains are agglomerated and partially separated in the matrix, with grains size being, on average, 26.7 nm. Cross-sectional imaging showed that the FePt grains have a truncated pyramid shape with a lower wetting angle, which is influenced by the surface energy of MgTiOBN. BN segregation at FePt grains or boundaries is still not clear. Using the electron energy loss spectrum (EELS), we found that part of the BN atoms were clearly observed in the FePt lattice and iron-boride oxide was indexed in the x-ray photoelectron spectroscopy (XPS) spectra. To determine the effects of BN segregant (from capping layer or intermediate layer) on the magnetic switching behavior of FePtCAg film, the intrinsic-(ΔH_int_ = 6.17 kOe, 6.54 kOe) and extrinsic- (ΔH_ext_ = 0.80 kOe, 0.39 kOe) switching field distribution (SFD) were measured by plotting saturated major- and unsaturated minor- hysteresis loops to evaluate the crystal orientation and microstructure (grains volume and distribution) for BN/FePtCAg/MgTiON and FePtCAg/MgTiOBN films, respectively. The main contribution of intrinsic SFD is the c-axis misalignment for the BN/FePt/MgTiON sample; however, the dispersed magnetic anisotropy has a higher input to intrinsic SFD for FePtCAg/MgTiOBN/CrRu film.

## 1. Introduction

Energy-assisted magnetic recording (MR), for example, heat-assisted MR, is a new technology that boosts hard disk area density beyond 2 Tb/in^2^. To extend the superparamagnetic effect of CoCrPt-(oxides) in perpendicular MR, higher magnetocrystalline anisotropy (K_u_) material, L1_0_ FePt film is optimal in this system [1,2,3,4]. The hard disk drive (HDD) based on FePt media has recently been tested in selected customers [5,6,7,8]. The intermediate layer has suitable lattice mismatch and provides a template for the growth of c-axis or (001) textured FePt film. The MgO-related materials, for example, using transition metal ion or nitride such as (Ti, Cu, Co, Ni, TiN) to replace part of the Mg^2+^ to improve the MgO target conductivity, have been discussed [9,10,11,12]. The surface energy and lattice misfit strain can be tuned between FePt and the intermediate layer and, finally, the FePt (00L) film can be sputtered on a (002) textured MgO-based intermediate layer using direct current magnetron sputtering.

The Ag, C, and BN are now the required multiple segregants for FePt media and amorphous BN is used to enhance columnar grains’ growth; the C has strong grains’ separation ability. Furthermore, the diffused Ag atoms which create vacancies at high temperatures were added to promote the order arrangement of Fe and Pt atoms in the lattice [6]. Based on our previous publications [11], we designed a C segregant that diffuses from the MoC_x_ intermediate layer, using a similar concept to determine the diffusion of BN from MgTiOBN. In addition, our previous work [13] found that the perpendicular anisotropy and coercivity of the FePtCAg film was enhanced significantly by the capped B_4_C layer due to diffusion and surface modification after high deposition temperature and the ultrathin BN capping layer was also considered to have a similar effect. As result, we deposited two kinds of layer sequences to compare BN segregant effect; the first is FePtCAg/MgTiOBN and the second is BN/FePtCAg/MgTiON (reference sample: extension of previous work [11,13]) on CrRu/glass. Furthermore, the (FePt/MgTiOBN) film illustrates lower saturation magnetization (M_s_) and magneto-crystalline anisotropy (K_u_) at a lower measurement temperature that deviates the normal tendency of the temperature dependence of the intrinsic magnetic properties mentioned above. To explain this result, the interface diffusion which causes the composition fluctuation was evidenced in microstructural grains mapping and elemental binding in the surface spectra. The theoretical simulations by density function theory (DFT) calculations assist the understanding of the structure–morphology–property relations for complex nanostructured systems of a similar nature and a similar level of complexity (e.g., containing transition metal atoms) [14,15,16].

We discuss the switching field distribution (SFD) of two FePtCAg samples. The separated and agglomerated grains were investigated to define the FePt grains size and magnetic clusters. The media noise can be reduced by lowering the intrinsic- and extrinsic-parts of SFD in the FePtCAg samples [4,17,18]. In this work, magnetic major and minor hysteresis loops were measured to define and understand the magnetic characteristics [4]. The intrinsic SFD contains a combination of dispersed magnetic anisotropy field, misaligned c-axis, and non-uniform grain size [18], and the extrinsic contribution belongs to the grains’ long-term dipole- and short-term exchange-coupling.

## 2. Materials and Methods

The FePtCAg/MgTiOBN and BN/FePtCAg/MgTiON samples were deposited by magnetron sputtering on the CrRu seed layer, which were also sputtered on a glass substrate (Corning, Eagle XG, Taipei, Taiwan). The substrate was cleaned in different solvents by ultrasonic vibration. The sputtering system was set up with the main- and pre-chamber and the substrate was transferred via the load-lock feedthrough to substrate holder, which has a heating up function by halogen lamp (OSRAM, 1000 W, GmbH, Munich, Germany) and the cathodes (AJA, A320, MA, USA) were set up on the main chamber.

The targets compositions used in this study are Cr_83_Ru_17_, (Mg_0.5_Ti_0.5_)O_0.9_N_0.1_, (Mg_0.5_Ti_0.5_)O_0.9_(BN)_0.1_, Fe_52_Pt_48_, Ag, C with 2 inches diameters. The CrRu seed layer was sputtered with a working pressure of 3 mTorr and the deposition rate was 0.165 nm/s. For MgTiON and MgTiOBN intermediate layers, the deposition was 0.045 nm/s, 0.036 nm/s, respectively and the Argon working pressure was 10 mTorr. For the magnetic FePt layer, the deposition rate was 0.088 nm/s, working pressure was 3 mTorr and the deposition rate of segregants CAg (40 vol%) was 0.035 nm/s.

The CrRu (90 nm) was direct-current sputtered at 217 °C on a glass substrate to determine the (002) texture and the MgTiON (002) or MgTiOBN (002) intermediate layer (30 nm) was deposited at 435 °C. The FePt (6 nm) magnetic layer was co-sputtered with 40 vol% (CAg) at 470 °C and BN (0.5 nm) was capped on the FePt reference film also at 470 °C. In this study, we deposited two kinds of layer sequences to compare BN segregant effect: first is FePtCAg/MgTiOBN and the second is BN/FePtCAg/MgTiON (reference sample: extension of previous work [11,13]) on CrRu/glass.

The crystal structure was performed by standard X-ray diffraction (XRD) (BRUKER, D8 Discover). The major- and minor- magnetic hysteresis loops were measured at varied temperatures by a superconducting quantum interference device magnetometer (SQUID, MPMS-XL, Quantum design, San Diago, CA, USA). The sample microstructure was observed by transmission electron microscopy (TEM, JEOL JEM-2010, Tokyo, Japan). The surface analysis was measured by X-ray photoelectron spectroscopy (XPS, ULVAC-PHI 5000, Hagisono, Chigasaki, Kanagawa, Japan).

## 3. Results and Discussion

**Figure 1:**
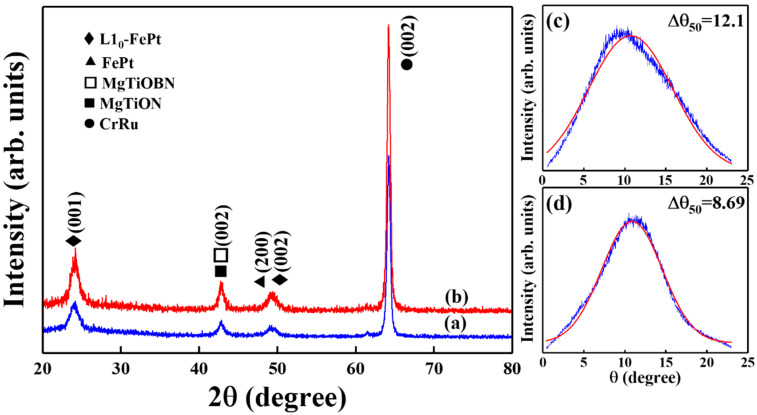
XRD patterns of (**a**) reference sample, (**b**) FePtCAg/MgTiOBN film and FePt(001) rocking curves of (**c**) reference sample, (**d**) FePtCAg/MgTiOBN films.

The crystal structure was investigated by XRD patterns in Figure 1 and the XRD patterns of reference and FePtCAg(6 nm)/MgTiOBN samples are presented in (a) and (b), respectively. The CrRu seed layer and the MgTiOX (X = N, BN) intermediate layers illustrate (002) texture in XRD patterns and the lattice misfit between MgTiOX (X = N, BN) and CrRu is 2.85% in (a), and 3.05% in (b). The (002) textured MgTiOX (X = N, BN)/CrRu underlayers promote the growth of (001) textured L1_0_ FePt. According to the structure data, the (001) superlattice peak appeared due to the ordered FePt phase, and order/disorder FePt (002) fundamental reflection peaks were also indexed.

Based on the FePt ordering degree equation published by B. E. Warren in reference [19,20], the ordering degree of (001) textured FePt film is proportional to the experimental integrated intensity ratio of FePt (001) and (002) reflection peaks, and the value of integrated intensity ratio [I(001)/I(002)] was changed from 2.82 to 2.59 in Figure 1a,b. However, the reflection peak indexed at 49.26° in Figure 1a (reference sample) is the overlapping of (200) peak at 48.94° which refers to the fct/fcc structure and (002) peak at 49.66°, which refers to ordered (L1_0_) phases. To consider the variant of “c”, “a” axis and the geometry of XRD, the Lorentz- and absorption-factors were corrected and the theoretical (I*_(002)_/I*_(001)_)^1/2^ value was estimated by thickness and rocking width [19,20]. In Figure 1b, the final ordering degree (I_(001)_/I_(002)_)^1/2^/(I*_(002)_/I*_(001)_)^1/2^ is 0.83. The c-axis deviated from the normal of FePtCAg thin film was measured by the rocking curve and the width at the half intensity of FePt (001) peaks are 12° and 8.7° in Figure 1c,d. The rocking curve of the reference sample presents higher c-axis misalignment due to the overlapped (200) and (002) peaks in Figure 1a. From the XRD pattern, the lattice constant of MgTiOBN is 0.422 nm estimated from the (002) reflection peak and the values for FePt film is a = 0.384 nm and c = 0.372 nm. The lattice mismatch is 3% between MgTiOBN/CrRu and 9% between FePt/MgTiOBN, respectively. The MgTiOBN(002)/[100] layer was grown epitaxially on the CrRu(002)/[110] seed layer and the FePtCAg film was grown on the MgTiOBN layer by hetero-epitaxial.

**Figure 2:**
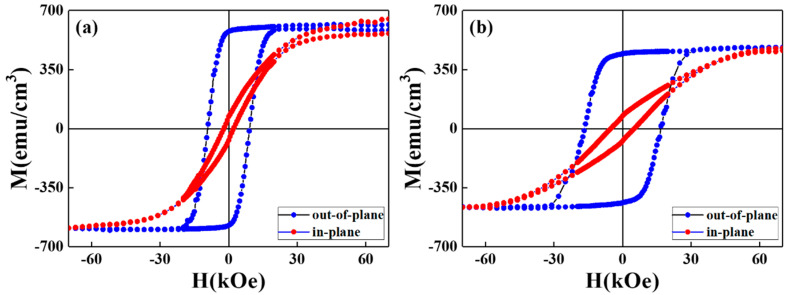
Easy-(out-of-plane) and hard-(in-plane) axis magnetization loops of (**a**) reference sample and (**b**) FePtCAg/MgTiOBN films measured at 350 K.

**Figure 3:**
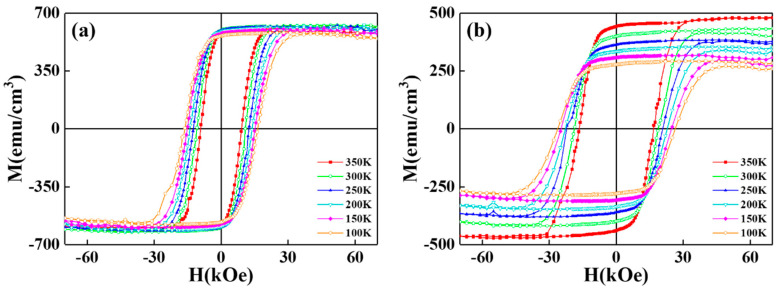
Easy-(out-of-plane) axis magnetization loops of (**a**) reference sample and (**b**) FePtCAg/MgTiOBN films measured from 100 K to 350 K.

Figure 2 shows the magnetic hysteresis loops in the easy- and hard- magnetization directions of reference and FePtCAg/MgTiOBN films measured at 350 K, and Figure 3 illustrates the perpendicular magnetization curves measured from 100 K to 350 K for two samples. The out-of-plane loops have a square-like shape with higher magnetization and a negative nucleation field, which defined at 0.95M_s_ and the in-plane loops are less wide. The maximum out-of-plane coercivity is 26 kOe for FePtCAg/MgTiOBN and 16kOe for the reference sample measured at 100 K.

**Figure 4:**
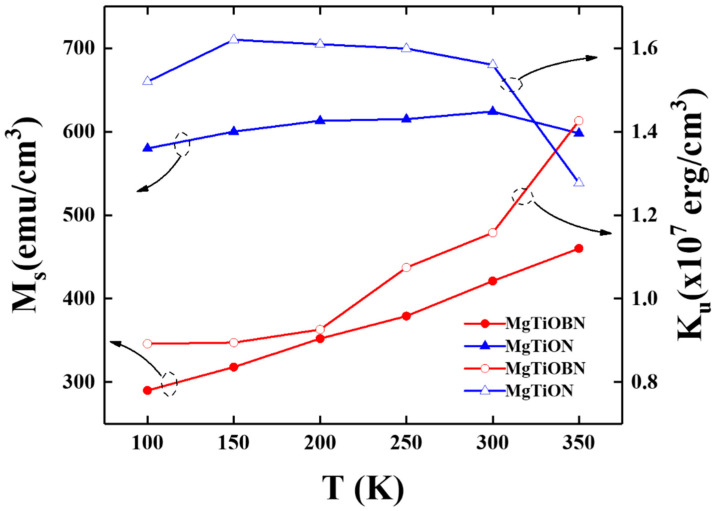
Temperature dependence of the saturation magnetization and magnetic anisotropy constant of reference sample and FePtCAg/MgTiOBN films.

Figure 4 presents the temperature dependence of the saturation magnetization (M_s)_ and K_u_ and the anisotropy field H_k_ equals to 2K_u_/M_s_). Here, H_k_ is determined by the intersection point of easy- and hard-axes magnetic hysteresis loops. The trend of saturation magnetization with varied measurement temperatures was quite different for the two samples. The saturation magnetization (Ms) of the reference sample is between 580 and 625 emu/cm^3^, measured from 100–350 K. However, the Ms value decreased significantly from 480 emu/cm^3^ to 290 emu/cm^3^ when the measured temperature changed from 350 K to 100 K for the FePtCAg/MgTiOBN film.

To understand the interface compositional fluctuation and avoid the thermal effect, the measured temperature was decreased from 350 K to 100 K. The variation in temperature dependence of M_s(_T) and K_u_(T) were found in two samples. The lower M_s_ in the reference sample was due to the diffused BN atoms from the MgTiOBN intermediate layer to the interstitial sites of the FePt lattice, as evidenced in the EELS spectrum.

MgO is a rock salt crystal structure, and the cations and anions are arranged in face centered cubic. The cation-anion radius ratio is between 0.414 and 0.732 and the coordination number for each ion is 6. The lattice constant of MgTiOBN is 0.422 nm, as estimated from XRD data in Figure 1, and the crystal radius is 0.0615 nm for Mg^2+^(Ti^4+^), 0.1492 nm for O^2−^, and 0.025–0.030 nm for BN ions. The BN crystal radius are much smaller than cations [Mg^2+^(Ti^4+^)] and are composed of interstitial impurity atoms.

After high temperature diffusion of BN atoms, there exist many defects and vacancies in the MgTiOBN intermediate layer. The interface Ti-Fe binding is stronger because FePt/TiO has a shorter bond distance than FePt/MgO [21]. As a result, less of TiN and TiON were bonded, and amorphous BN may also form in the interstitial sites in MgTiOBN intermediate layer. The stronger Ti-Fe binding enhanced the spin-down electrons, and the net magnetic moment (or M_s_) was reduced [21].

**Figure 5:**
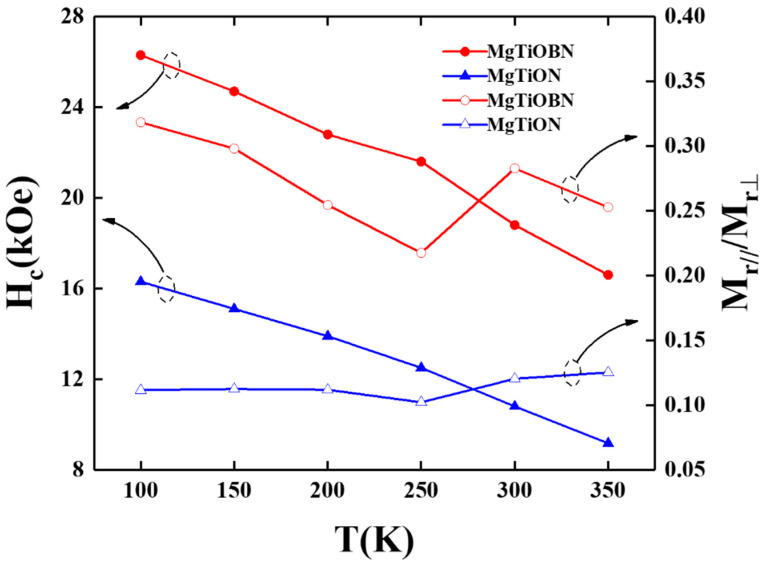
Temperature dependence of coercivity (normal to film surface) and remanence ratio of reference sample and FePtCAg/MgTiOBN films.

When the measured temperature is lower, both the thermal effect of Ti-Fe binding and net magnetic moment (or M_s_) are smaller and demonstrate a similar trend to K_u_(T). The temperature dependence of the out-of-plane coercivity and the ratio of parallel-(M_r(in-plane)_) to perpendicular-(M_r(out-of-plane)_) magnetization are shown in Figure 5. The out-of-plane H_c_ increased when the measured temperature decreased from 350 K to 100 K and the H_c_ value of the reference sample is around 10 kOe smaller than FePtCAg/MgTiOBN film. The disordered soft- and plane variant hard-magnetic phases [22,23] induced the in-plane magnetization components and out-of-plane H_c_ deterioration. The FePt grains were most aligned in the c-axis(easy-magnetization) direction and the remanence ratio ranged from 0.1 to 0.3.

**Figure 6:**
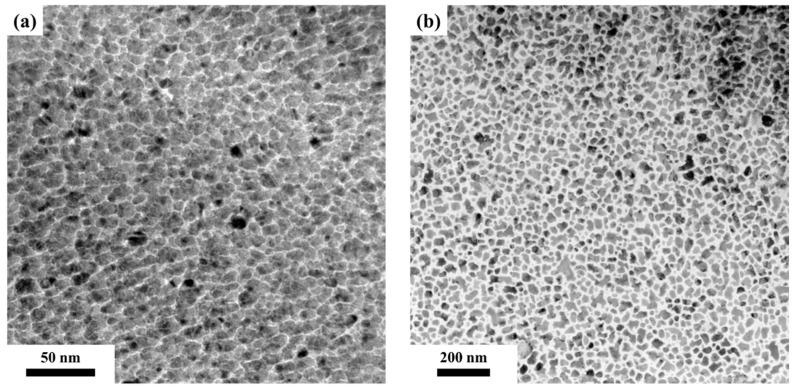
The plane-view TEM images of (**a**) reference sample and (**b**) FePtCAg/MgTiOBN/CrRu film.

**Figure 7:**
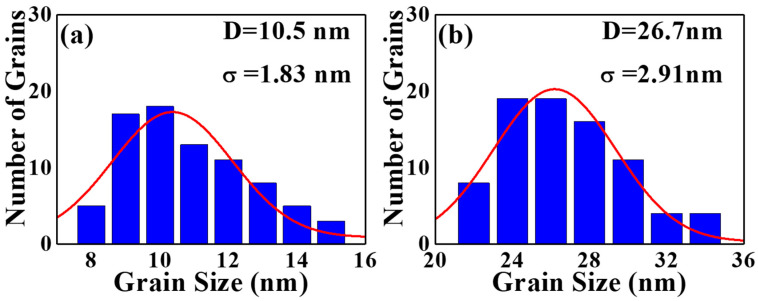
The grain size distribution and fitted average grains size of (**a**) reference sample and (**b**) FePtCAg/MgTiOBN film.

The microstructure of the FePtCAg film is shown in the plane-view TEM images in Figure 6a reference sample and Figure 6b FePtCAg/MgTiOBN film, and Figure 7 presents grain size distribution. After high-temperature deposition, the diffused BN was segregated at the grains boundary or into the FePt lattice. The average grain size is 10.5 nm, as illustrated in Figure 6a and Figure 7a. However, many FePt grains were still interconnected in a worm like-shape. In Figure 6b, the BN was also diffused from the MgTiOBN intermediate layer to the FePtCAg layer, but a BN compound was observed in the FePt lattice, as evidenced in the EEL spectrum. As a result, in Figure 6b and Figure 7b, the FePt grains are coarsened with grain sizes of 26.7 nm, which implies higher crystallinity. This result is consistent with the XRD result; the FePtCAg film presents higher crystallinity on the MgTiOBN layer, which shows higher crystallinity than the MgTiON layer and the crystallinity of CrRu seed layer is almost the same for the two samples.

**Figure 8:**
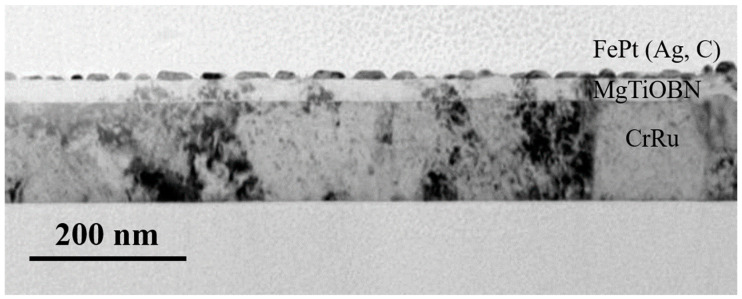
Cross-section TEM image of FePtCAg/MgTiOBN/CrRu films.

Figure 8 illustrates the cross-sectional grains morphology of FePtCAg/MgTiOBN film and the FePt grains are separated in the structure of islands on continuous MgTiOBN/CrRu films. The wetting angles at two sides of islands range from 35 to 67°, resulting in the close surface energy between FePt(001) and MgTiOBN(002).

**Figure 9:**
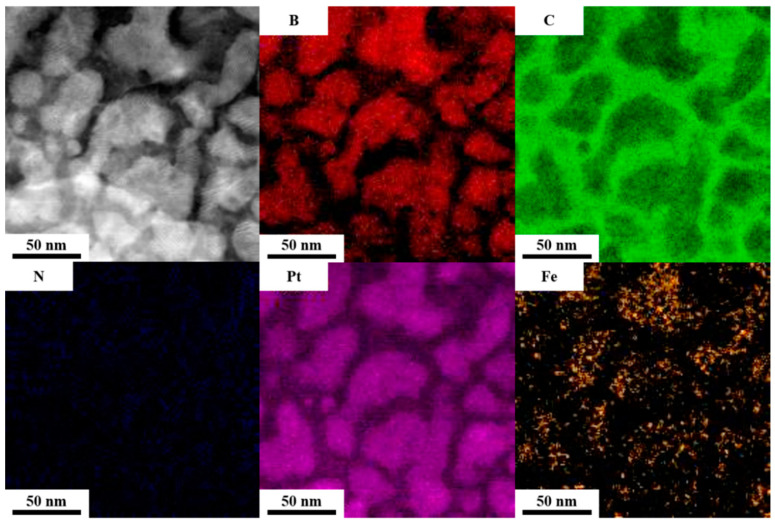
EELS mapping of Fe, Pt, B, N, C elements by HRTEM.

Figure 9 shows the FePt, BN, C mapping by the electron energy loss spectroscopy (EELS) which is useful to detect the light elemental atoms. To compare to the plane view image (zero loss images), the Fe, Pt elements are located in the interconnected (or agglomerated) islands areas which are FePt grains.

**Figure 10:**
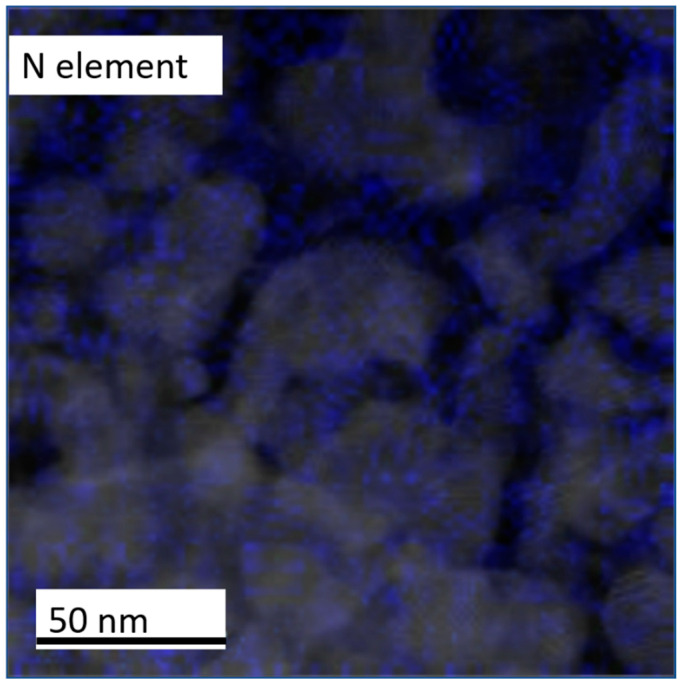
EELS mapping of grains image and N elements (indigo color).

The C atoms are almost distributed in the grain boundary area and the BN atoms are both clearly observed in the FePt lattice. This was monitored by overlapped TEM images in Figure 9 and we found that the N atoms are located in the FePt grains and part of them are distributed in the boundaries. To observe the N distribution clearly, the N image was mapped with the FePtCAg grains image presented in Figure 10. The composition and elements distribution are summarized into two parts, (1) The BN was truly diffused up from the MgTiOBN intermediate layer to the FePt lattice and part of them are formed in amorphous BN binding and the other BN atoms are bonded to Fe atoms around the grains shell. (2) The C atoms are almost segregated at the FePt grains boundaries. To further understand the other elements’ distribution in FePtCAg/MgTiOBN film, the energy dispersive X-ray (EDX) was applied to analyze the composition. 

**Figure 11:**
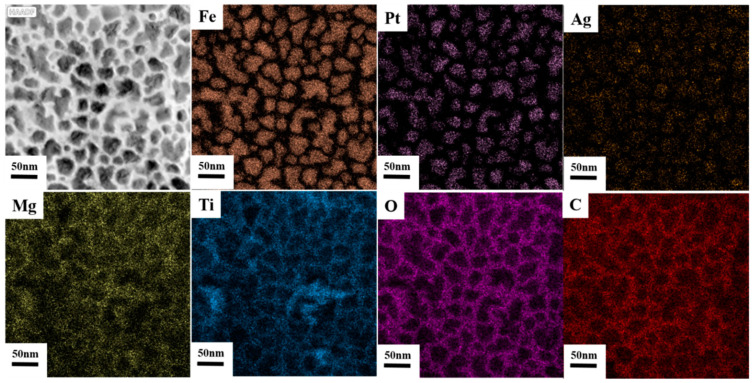
High angle angular dark field (HAADF) image and EDX mapping of Fe, Pt, Ag, Mg, Ti, O, C elements.

Figure 11 shows the plane view elements distribution of FePtCAg/MgTiOBN film. Both Fe and Pt elements appear in the grain areas with the same position. Part of the Ag element was detected in the FePt grain areas. Mg, Ti, O and C elements appear in the grain boundary areas. The signal of B and N elements are not detected because EDX mapping is more effective in detecting heavier atoms.

**Figure 12:**
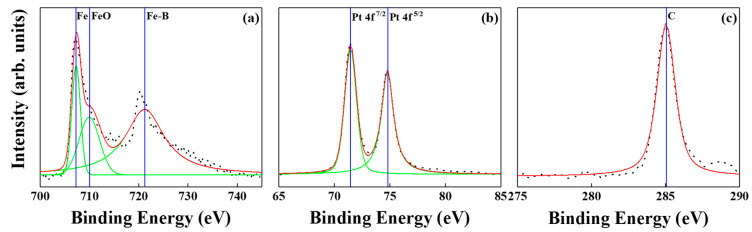
XPS spectra of BN/FePtCAg/MgTiON film, (**a**) the metallic Fe, iron-oxide and iron-boride are indexed, (**b**) Pt and (**c**) C.

**Figure 13:**
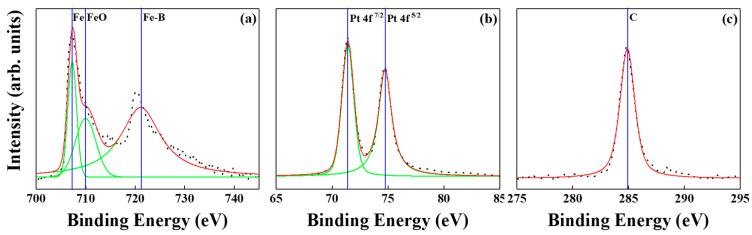
XPS spectra of FePtCAg/MgTiOBN film, (**a**) the metallic Fe, iron-oxide and iron-boride are indexed, (**b**) Pt and (**c**) C.

Figure 12 and Figure 13 show the XPS spectra of BN/FePtCAg/MgTiON and FePtCAg/MgTiOBN films. The iron-oxide and -boride were detected after peaks resolution and metallic Pt; C peaks were also checked. Based on the microstructural and surface analysis, the partially separated FePt grains by segregant C atom were not enough to interrupt the lateral grains interconnecting after the sputtering process, and BN was diffused from the intermediate layer to the interstitial site of the FePt lattice and formed the Fe-B, Fe-N, B-N bindings. From the XPS report [9], it was seen that titanium oxides [TiO (−542.7 kJ/mol), TiO_2_ (−849.1 kJ/mol)] are more favorable than TiN (−337.7 kJ/mole), based on the formation enthalpy [24]. From EELS mapping, the N atoms appeared both in the FePt lattice and grains boundaries. This is because the N atoms were easy to reject from the lattice and created vacancies and defects inside the FePt lattice, which enhance ordering and coercivity [25,26].

In conclusion, the FePtCAg deposited on MgTiOBN illustrates lower K_u_(T) and M_s_(T), however, the crystallinity is higher, as evidenced in the smaller rocking width (less c-axis misalignment) and larger average grain size. Furthermore, the FePt(Ag, C)/MgTiOBN film presents more grain separation, which was observed in the wider grains boundaries (or larger grains pitch) in the plane-view TEM image. It is supposed that the magnetic rotation was dominant in more separated FePt grains, which usually have larger H_c_ (normal to film surface) than (worm-like shape) FePt grains in the reference sample. The domain wall motion behavior was preferred in partially interconnected FePt grains.

**Figure 14:**
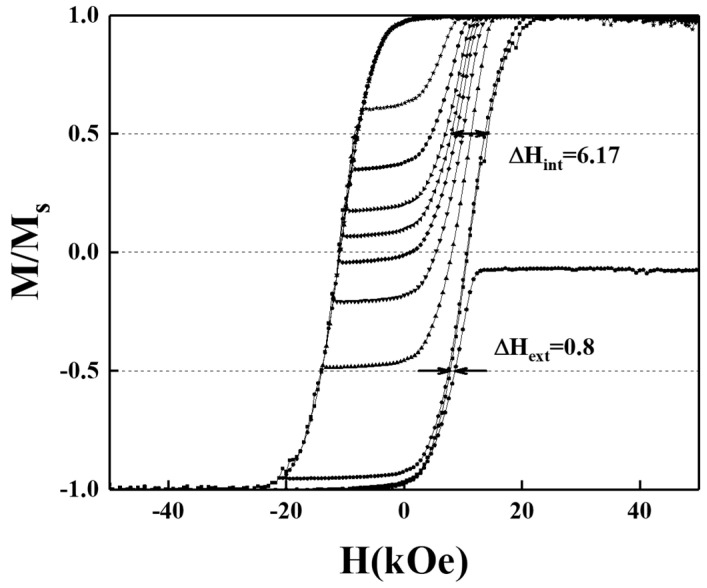
Magnetization curves include major and minor hysteresis loops of the BN/FePtCAg/MgTiON film. Switching field distribution was evaluated by measured loops.

**Figure 15:**
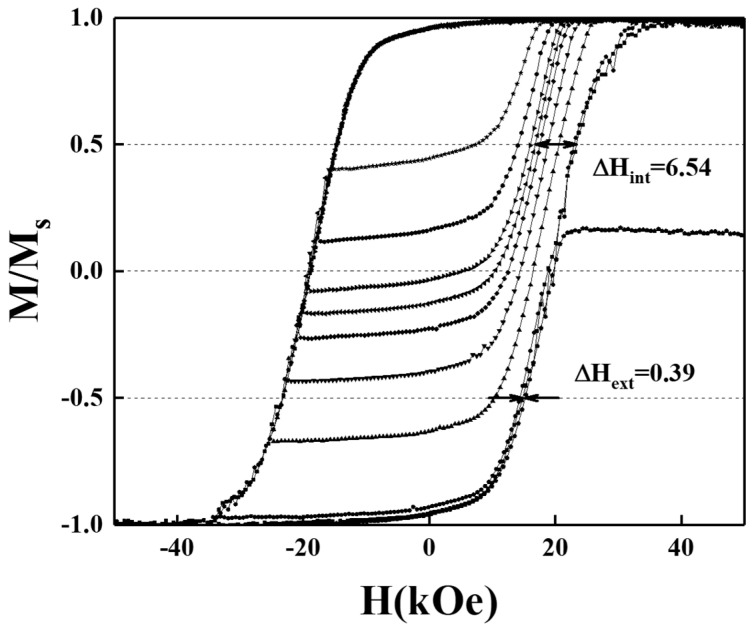
Magnetization curves include major and minor hysteresis loops of the FePtCAg/MgTiOBN film. Switching field distribution was evaluated by measured loops.

To determine the correlation between the magnetization reversal process and crystal structure (c-axis orientation) and microstructure (grains size), we analyzed the intrinsic and extrinsic parts of the switching field distribution (SFD) of the FePt grains. Figure 14 and Figure 15 present the magnetization curves, including the minor- and major- hysteresis loops of BN/FePtCAg/MgTiON and FePtCAg/MgTiOBN samples and the intrinsic-(ΔH_int_ ), and extrinsic-(ΔH_ext_) switching field distribution were collected from the minor loops. When the field of recoil loop increased up to the coercivity field, 50% of the FePt grains on the easy axis switched and the magnetostatic interaction between grains was not considered. The grain size variation (σ_volume_), the magnetocrystalline anisotropy deviation (σ_Hk_), and the c-axis(σ_axis_) misalignment belong to intrinsic SFD and grains coupling (dipolar- or exchange-interaction) is extrinsic SFD [4,17,27,28,29]. The dimension, structure- and magnetic- alignment of each grain was discussed in ΔH_int_ and the interaction between many grains was considered in ΔH_ext_. For the granular structure, ΔH_int_ focus on the uniformity of the properties of each grain and ΔH_ext_ discusses the short- and long-term interactions of grains. The standard deviation (σ_int_) is 6.17 kOe for the BN/FePtCAg/MgTiON sample and 6.54 kOe for FePtCAg/MgTiOBN film, and the (σ_int_)^2^ equals to (σ_volume_)^2^ + (σ_axis_)^2^ + (σ_Hk_)^2^.

The (σ_axis_) was Gaussian fitting the FePt(001) rocking curve in Figure 1c,d and the values are 5.14kOe and 3.69kOe for the respective reference and FePtCAg/MgTiOBN samples [13]. The (σ_volume_) was obtained after fitting the magnetization reversal model in Equation (1) [14] and the values are 0.35 kOe and 0.73 kOe, respectively.

2M(H) = Erfc(x)

(1) $$x=\left\{\sigma_{v}^{2}-ln\left[\left(\frac{1}{V}\right)ln\left(\frac{f_{0}t}{ln2}\right)\left(\frac{k_{B}T}{K_{u}}\right)\left[\frac{1}{{\left(1-\frac{HM_{s}}{2cK_{u}}\right)}^{2}}\right]\right]\right\}\left[\frac{1}{\sqrt{2\sigma_{v}}}\right]$$

The magnetic anisotropy deviation (σ_Hk_)^2^ was obtained from (σ_int_)^2^ − (σ_volume_)^2^ − (σ_axis_)^2^ and the values of (σ_Hk_) are 3.39 kOe and 5.35 kOe for the respective two samples. The main contribution of intrinsic SFD is the c-axis misalignment for the reference sample, however, the FePtCAg/MgTiOBN film has a higher magnetic anisotropy deviation. Magnetostatic- and exchange-coupling comes from interconnected magnetic grains [4,27] and the magnetic cluster size (D_n_) was estimated by Equation (2), which is approximated from [21].

H_ext_ = H_in_ ~ H_ms-long_(D_n_) = −[1+(D_n_/t_mag_)^2^]^−^^1/2^M_mag_ (2)

From Equation (2), the internal effective field (H_in_) for magnetic grains [18] was approximated to a long-range magnetostatic field which was considered as the main contribution of extrinsic SFD. Compared to the microstructural average grains size, 10.5 nm and 26.7 nm, the magnetic cluster size is 118 nm and 79 nm for the respective two samples [13]. The magnetization reversal parameters measured by minor- and major-loops of the reference sample and FePtCAg/MgTiOBN film are listed in Table 1. The thermal stability was estimated by (K_u_V/k_B_T) at 300K and the values were 198 and 1110 for the reference sample and the FePtCAg/MgTiOBN film, respectively. These values are much higher than the required number 60.

## 4. Conclusions

The analysis of switching field distribution in BN/FePtCAg/MgTiON and FePtCAg/MgTiOBN films is consistent with the experimental crystal orientation and microstructural grains results. For the reference sample, the main contribution of intrinsic SFD is the c-axis misalignment and the higher extrinsic SFD is due to more grain interactions, evidenced in the interconnected worm-like FePt grains. However, the FePtCAg/MgTiOBN film has a higher magnetic anisotropy deviation due to the compositional fluctuation at the interface, which causes a decrease in saturation magnetization, and the extrinsic SFD is lower, as evidenced in the thick grain boundary areas.

## Figures and Tables

**Table 1:** Magnetic switching field behaviors of FePtCAg/MgTiOBN film and BN/FePtCAg/MgTiON (reference sample).

**Sample**	**ΔH_int_ (kOe)**	**σ_axis_ (kOe)**	**σ_vol_ (kOe)**	**σH_k_ (kOe)**
BN/FePt/MgTiON	6.17	5.14	0.37	3.39
FePt/MgTiOBN	6.54	3.69	0.73	5.35
**Sample**	**σ_int_ (kOe)**	**Δ** **H_ext_ (kOe)**	**Cluster Size** **(nm)**	**Grain Size** **(nm)**
BN/FePt/MgTiON	6.17	0.80	118	10.5
FePt/MgTiOBN	6.54	0.39	79	26.7

## Data Availability

Data are contained within the article.
